# Supplementary material for: HIV-1 gp120-induced lysosomal stress responses are controlled by TRPML1 redox sensors
Source: Redox Rep. 2026 Jun 26;31(1):2679333. doi: 10.1080/13510002.2026.2679333 (PMC13312838; doi:10.1080/13510002.2026.2679333)
Supplement: Supplementary Material — Supplementary data_clean copy.docx [file YRER_A_2679333_SM5163.docx]

**Supplementary Materials**

**HIV-1 gp120-induced lysosomal stress responses are controlled by TRPML1 redox sensors**

Nirmal Kumar^a,b^, Braelyn Liang^a^, and Jonathan D. Geiger^a,^*

^a^Department of Biomedical Sciences, University of North Dakota School of Medicine and Health Sciences, Grand Forks, North Dakota, 58203, USA

^b^Department of Neuroscience and Cell Biology, Rutgers University Robert Wood Johnson Medical School, Piscataway, New Jersey, USA

***Corresponding author:**

Jonathan D. Geiger, Ph.D.

Chester Fritz Distinguished Professor

Department of Biomedical Sciences

University of North Dakota School of Medicine and Health Sciences

504 Hamline Street, Room 110

Grand Forks, ND 58203

(701) 777-2183; jonathan.geiger@und.edu

**The document includes:**

Supplementary Fig.1 to 5


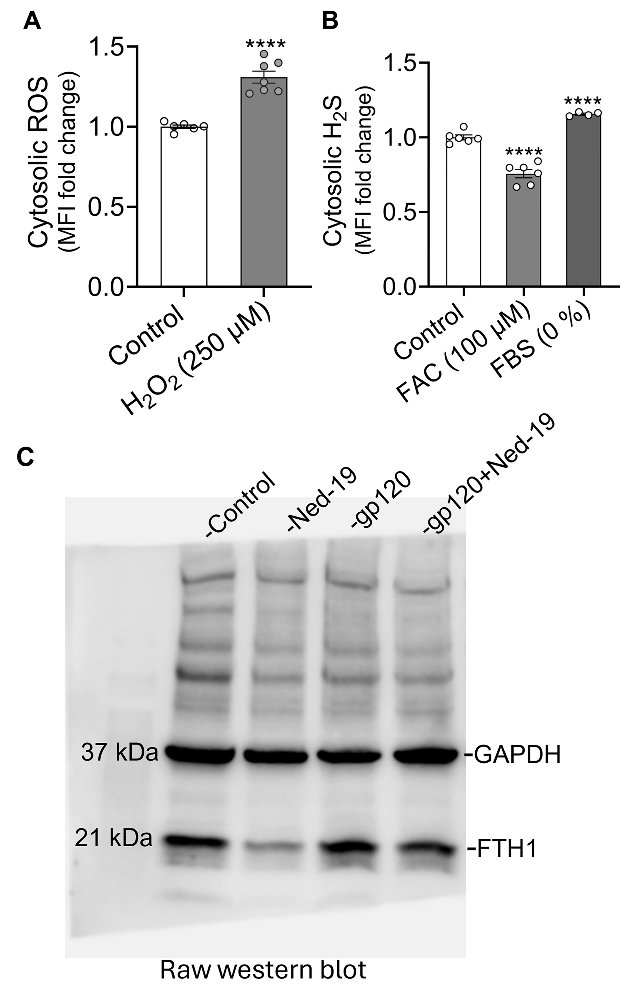


**Supplementary Fig. 1:** H_2_O_2_ increased cytosolic ROS, serum deprivation increased cytosolic H_2_S, and FAC decreased cytosolic H_2_S. **(A)** SH-SY5Y cells were treated with H_2_O_2_ (250 μM, 4 h) or vehicle control, stained with CM-H_2_DCFDA, and fluorescence was measured by flow cytometry. H_2_O_2_ significantly increased cytosolic ROS levels. **(B)** SH-SY5Y cells were treated with FAC (100 μM, 4 h), vehicle control or incubated in FBS-free medium (0% FBS, 4 h) versus complete medium. Cells were stained with SF7-AM to detect H_2_S and fluorescence was measured by flow cytometry. FAC significantly decreased cytosolic H_2_S levels, whereas serum deprivation significantly increased H_2_S. **(C)** Representative uncropped raw western blot image corresponding to the cropped blot shown in main Fig. 1D. Data are shown as mean ± SEM with individual data points (n = 6-7). Two-tailed Student’s *t*-test or one-way ANOVA with Tukey’s multiple comparison was used for statistical analysis. *****p*< 0.0001


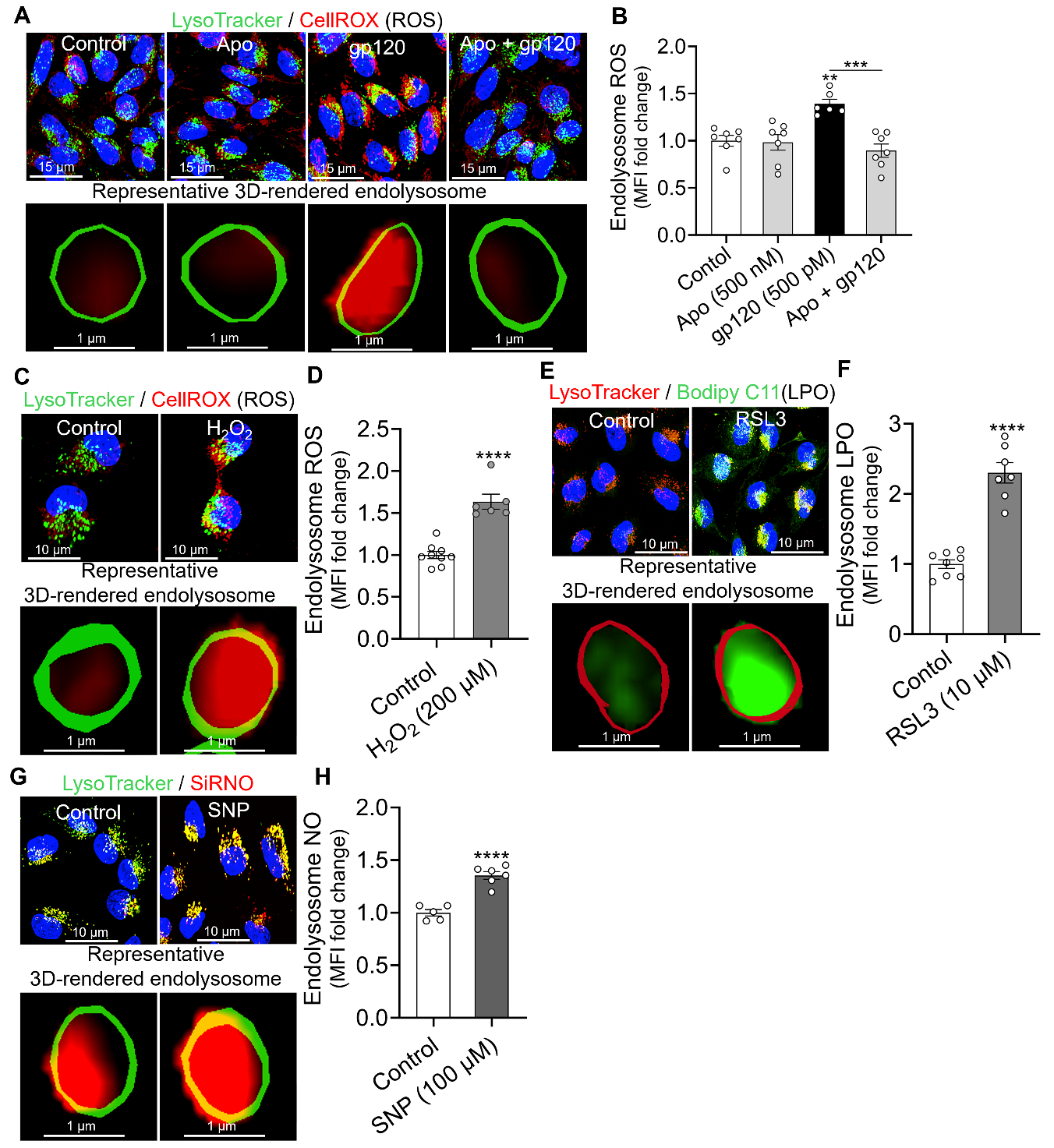


**Supplementary Fig. 2:** gp120-induced increases in endolysosome ROS were blocked by NOX2 inhibition, and different positive controls increased endolysosome ROS, lipid peroxidation and nitric oxide. **(A)** Representative confocal images of SH-SY5Y cells pretreated with the NOX2 inhibitor apocynin (500 nM, 30 min) or vehicle (control) and then treated with gp120 (500 pM, 4 h). Cells were stained with CellROX (red) for ROS, LysoTracker Green (green) for endolysosome, and Hoechst 33342 (blue) for nuclei. Scale bars = 10 µm. A representative Imaris-generated surface rendering of a Lysotracker-positive endolysosome containing ROS fluorescence is also shown. Scale bar = 1 µm. **(B)** quantification of CellROX fluorescence in LysoTracker-positive endolysosomes. gp120-induced increases in endolysosome ROS were blocked by apocynin. **(C-E)** Representative confocal images of SH-SY5Y cells treated with H_2_O_2_ (200 μM, 4 h), RSL3 (10 μM, 4 h), SNP (100 μM, 4 h) or vehicle (control). Cells were stained with stained with CellROX (red) for ROS, Bodipy C11(green) for lipid peroxidation (LPO), or siRNO (red) for nitric oxide (NO), along with LysoTracker Green or Red for endolysosomes, and Hoechst 33342 (blue) for nuclei. Scale bars = 10 µm. Representative Imaris-generated Lysotracker-positive endolysosome containing ROS, LPO, or NO fluorescence are also shown. Scale bar = 1 µm. **(F-H)** Quantification of CellROX or Bodipy C11, or SiRNO fluorescence in LysoTracker-positive endolysosomes. H_2_O_2_ increased endolysosome ROS, RSL3 increased LPO, and SNP increased NO. Data are shown as mean ± SEM with individual data points (n = 5-9). Each data point represents MFI in LysoTracker-positive endolysosomes from at least 50 cells per condition. Statistical analysis was performed using Two-way ANOVA with Tukey’s multiple comparison or two-tailed Student’s *t*-test. ***p*< 0.01, ****p*< 0.001, *****p*< 0.0001


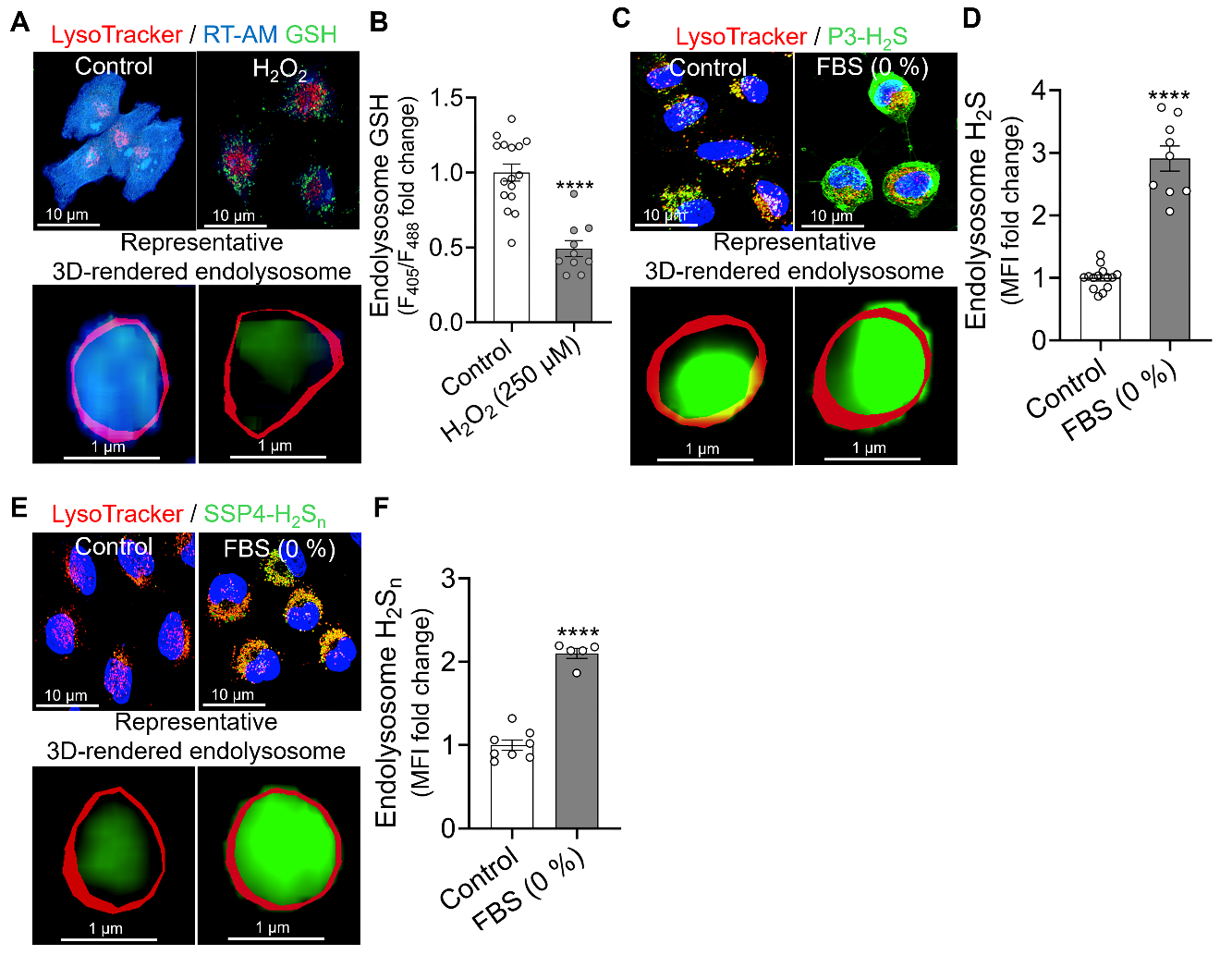


**Supplementary Fig. 3:** H_2_O_2_ decreases endolysosome GSH, and serum deprivation elevates H_2_S and sulfane sulfur. (**A,C,E)** Representative confocal images of SH-SY5Y cells treated with H_2_O_2_ (250 µM, 1 h) or vehicle control or incubated in FBS-free medium (0% FBS, 4 h) versus complete medium (control). Cells were stained with RT-AM GSH (blue/green) for GSH, P3 (green) for H_2_S or SSP4 (green) for sulfane sulfur (H_2_S_n_). Endolysosomes were labeled with LysoTracker Far Red (red) or LysoTracker Red, and nuclei with Hoechst 33342 (blue). Scale bars = 10 µm. A representative Imaris-generated surface rendering of a Lysotracker-positive endolysosome containing GSH , H_2_S, or H_2_S_n_ fluorescence are also shown. Scale bar = 1 µm. **(B,D,F)** Measurement of RT-AM ratio (B), P3 (D), or SSP4 (F) fluorescence within LysoTracker-positive endolysosomes. Data are presented as fold change of mean fluorescence intensity (MFI). H_2_O_2_ significantly decreased endolysosome GSH, while serum deprivation increased endolysosome H_2_S and H_2_S_n_. Data are shown as mean ± SEM with individual data points (n = 5-16). Each data point represents the MFI within LysoTracker-positive endolysosomes from a minimum of 100 cells per condition. Two-tailed Student’s *t*-test was used. *****p*< 0.0001


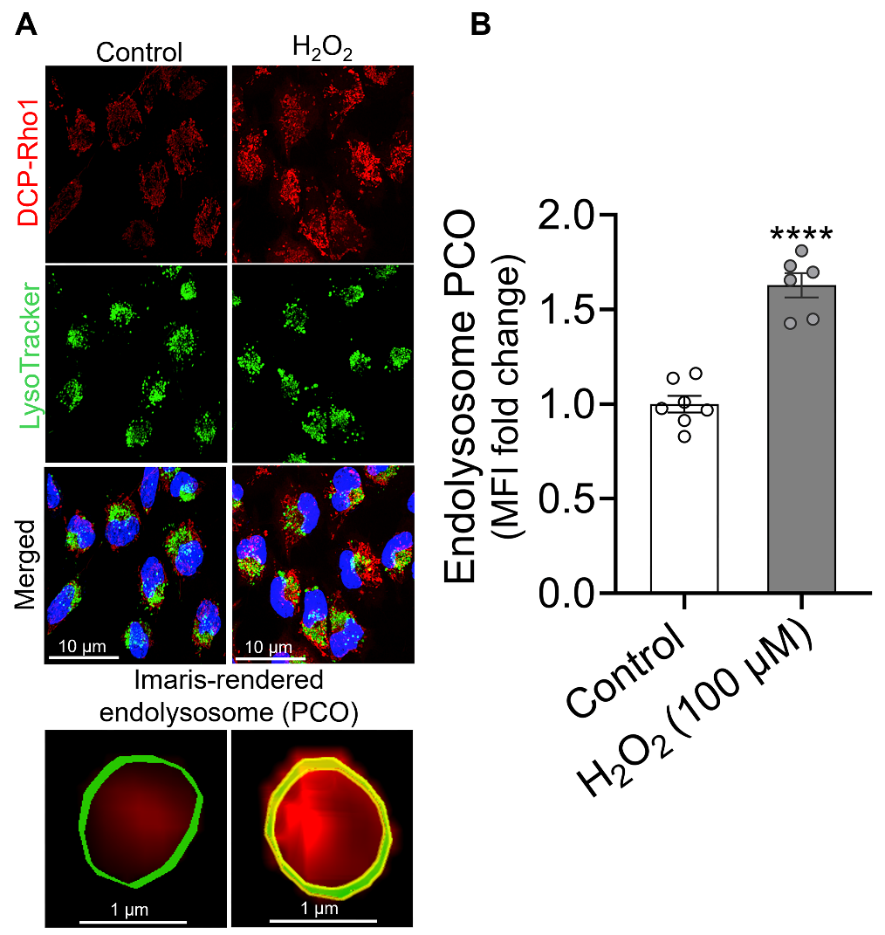


**Supplementary Fig. 4:** H_2_O_2_ increases protein cysteine oxidation (PCO) in endolysosome proteins. **(A)** Representative confocal images of SH-SY5Y cells treated with H_2_O_2_ (100 µM, 1 h) or vehicle (control) and stained with DCP-Rho1 (red) for protein cysteine oxidation (PCO), LysoTracker Green (green) for endolysosomes, and Hoechst 33342 (blue) for nuclei. Scale bars = 10 µm. A representative Imaris-generated surface rendering of a Lysotracker-positive endolysosome containing DCP-Rho1 fluorescence are also shown. Scale bar = 1 µm. **(B)** Measurement of DCP-Rho1 fluorescence within LysoTracker-positive endolysosomes using Imaris software. Data are shown as fold change of mean fluorescence intensity (MFI). H_2_O_2_ significantly increased endolysosome PCO. Data are presented as mean ± SEM with individual data points (n = 6-7). Each data point represents DCP-Rho1 MFI within LysoTracker-positive endolysosomes from a minimum of 100 cells per condition. Two-tailed Student’s *t*-test was used for statistical analyses. *****p*< 0.0001


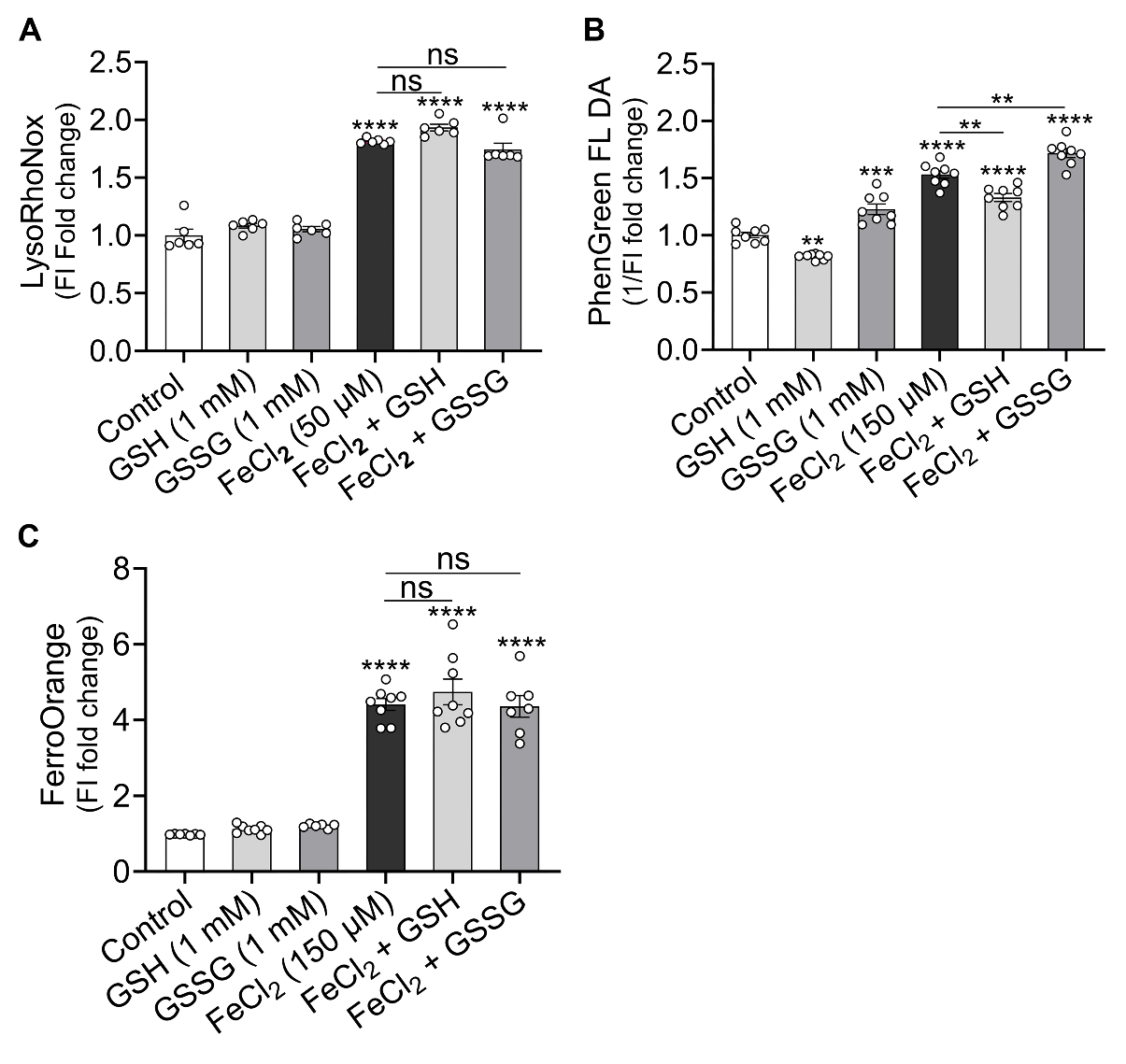


**Supplementary Fig. 5:** Effect of GSH and GSSG on Fe^2+^ detection by fluorescent probes. LysoRhoNox (5 μM), PhenGreen FL DA (5 μM), or FerroOrange (1 μM) was diluted in PBS in 96-well plates, with or without GSH (1 mM) or GSSG (1 mM). FeCl_2_ (50, or 150 μM) was then added, and fluorescence was measured using a microplate reader. Data are presented as fold change in fluorescence intensity. **(A)** LysoRhoNox fluorescence intensity (FI) was increased by FeCl_2_ (50 μM) but not altered by GSH (1 mM) or GSSG (1 mM) alone or in combination with FeCl_2_. **(B)** Reciprocal PhenGreen FI (1/FI) was increased by FeCl_2_ (150 μM). GSH decreased whereas GSSG increased 1/FI values, both alone and with FeCl_2_. **(C)** FerroOrange FI was increased by FeCl_2_ (150 μM) but was unaffected by GSH or GSSG alone or in combination with FeCl_2_. Data are shown as mean ± SEM with individual data points (n = 6-8). Each data point represents the fluorescence intensity from one well of a 96-well plate. Two-way ANOVA with Tukey’s multiple comparison was used. ns = non-significant, ***p*<0.01, ****p*<0.001, *****p*<0.0001
